# Studying Upper-Limb Kinematics Using Inertial Sensors Embedded in Mobile Phones

**DOI:** 10.2196/rehab.4101

**Published:** 2015-05-20

**Authors:** Cristina Roldan-Jimenez, Antonio Cuesta-Vargas, Paul Bennett

**Affiliations:** ^1^Departamento de Psiquiatría y FisioterapiaFacultad de Ciencias de la Salud, Andalucia Tech, Cátedra de Fisioterapia y Discapacidad, Instituto de Biomedicina de Málaga (IBIMA), Grupo de Clinimetria (FE-14)Universidad de MálagaMalagaSpain; ^2^School of Clinical ScienceQueensland University of TechnologyBrisbaneAustralia

**Keywords:** patient outcome assessment, shoulder, upper extremity, kinematics

## Abstract

**Background:**

In recent years, there has been a great interest in analyzing upper-limb kinematics. Inertial measurement with mobile phones is a convenient and portable analysis method for studying humerus kinematics in terms of angular mobility and linear acceleration.

**Objective:**

The aim of this analysis was to study upper-limb kinematics via mobile phones through six physical properties that correspond to angular mobility and acceleration in the three axes of space.

**Methods:**

This cross-sectional study recruited healthy young adult subjects. Humerus kinematics was studied in 10 young adults with the iPhone4. They performed flexion and abduction analytical tasks. Mobility angle and lineal acceleration in each of its axes (yaw, pitch, and roll) were obtained with the iPhone4. This device was placed on the right half of the body of each subject, in the middle third of the humerus, slightly posterior. Descriptive statistics were calculated.

**Results:**

Descriptive graphics of analytical tasks performed were obtained. The biggest range of motion was found in pitch angle, and the biggest acceleration was found in the y-axis in both analytical tasks. Focusing on tridimensional kinematics, bigger range of motion and acceleration was found in abduction (209.69 degrees and 23.31 degrees per second respectively). Also, very strong correlation was found between angular mobility and linear acceleration in abduction (*r*=.845) and flexion (*r*=.860).

**Conclusions:**

The use of an iPhone for humerus tridimensional kinematics is feasible. This supports use of the mobile phone as a device to analyze upper-limb kinematics and to facilitate the evaluation of the patient.

## Introduction

Upper-limb mobility is of great interest in clinical settings [1] because measuring the range of motion (ROM) is critical when evaluating the musculoskeletal system [2]. Upper extremities have been measured by manual goniometry for the last 100 years, but measurement methods have recently expanded [3-6]. Besides goniometry, arm ROM has been studied by other methods, such a digital goniometer [5], visual estimation [7], digital inclinometer [8,9], three-dimensional (3D) gyroscope [10,11], polhemus fastrak [12], calibration anatomical system techniques [13], the Kinect system [14], biplane fluoroscopy [15,16], markers fitted on intracortical pins [17], 3D computerized tomography [18], and the moiré fringe projection technique [19].

Recently, telerehabilitation has provided rehabilitation using Internet communication as a result of emerging contemporary technologies for therapeutic purposes [20,21]. Thus, Internet-based evaluation and goniometry have been accepted as new, valid, and reliable tools for measuring ROM [22]. This drives the use of the mobile phone as a tool for assessing and measuring. Mobile phone apps are being validated as goniometric tools [23] through clinometers [24] or goniometers [25]. Image-based apps have been created for measuring elbow and hallux valgus angles [26,27], and clinometer-based apps have also been created for measuring shoulder ROM [24]. In addition, an inclinometer-based app on a mobile phone has been demonstrated to have an acceptable reliability compared to conventional inclinometers that evaluate the shoulder joint [28]. Furthermore, active shoulder external rotation measures have been validated using inclinometery-based and image-based apps [29]. Recently, a study has analyzed arm motion by inertial variables provided by a mobile phone in five subjects [30].

One of the recently used techniques has been inertial sensors. Their use in human analysis involves a valid and reliable method that provides the potential required for dynamic 3D motion analysis [31]. Their protocol [32] and intra- and interoperator reliability [33] in the upper extremity have been determined. In addition, their operational feasibility in various clinical applications has been studied [34]. Several protocols have also been developed for analyzing the scapulothoracic, humerothoracic, and elbow joints [35], as well as scapula [36]. Very recently, reliability and precision of scapula kinematic through inertial and magnetic measurement systems (IMMS) has been studied in healthy subjects [37]. Advantages and disadvantages of these sensors have been discussed as part of a variety of motion analysis systems [38]. Thus, inertial sensors embedded in mobile phones have been used for analyzing movement, such as trunk kinematics [39]. More specifically, they have been used for evaluating shoulder movement using kinematic scores to assess the difference between healthy and painful shoulders [30].

Emerging mobile phone use for therapeutic purposes [40] has led to the need for research on arm ROM using mobile phones while incorporating the qualities of inertial sensors that allow clinicians an inexpensive and easy-to-use tool for upper extremity evaluation and outcome assessment.

The purpose of this study was to study humerus kinematics through two physical properties that correspond to angular mobility and acceleration in the three axes of space, obtained by inertial sensors embedded in a mobile phone.

## Methods

### Subjects

This cross-sectional study recruited healthy young adults from the Faculty of Health Sciences (University of Málaga) who were interested in taking part in the project. Subjects provided inclusion and exclusion criteria. Inclusion criteria included being aged between 18 and 35 years, having a Body Mass Index (BMI) between 18.5 and 28, and being right-handed. Exclusion criteria included consuming analgesics or non-steroidal anti-inflammatory drugs (NSAIDs) and suffering from shoulder pathology.

Ten subjects (7 men and 3 women) were included. Mean age was 24.2 years (SD 4.04 years), and average BMI was 22.59 kg/m^2^(SD 2.4 kg/m^2^; see Table 1).

The ethics committee of the University of Málaga, Spain, approved this study. Written consent was obtained following an explanation of the procedures.

**Table 1:** Values of anthropometric and descriptive variables.

	Minimum	Maximum	Mean	Standard deviation
Age, years	20.00	34.00	24.20	4.04
Size, cm	156.00	184.00	172.20	9.05
Weight, kg	48.00	87.00	66.60	11.88
BMI, kg/m^2^	19.72	27.46	22.59	2.40

### Apparatus

**Figure 1:**
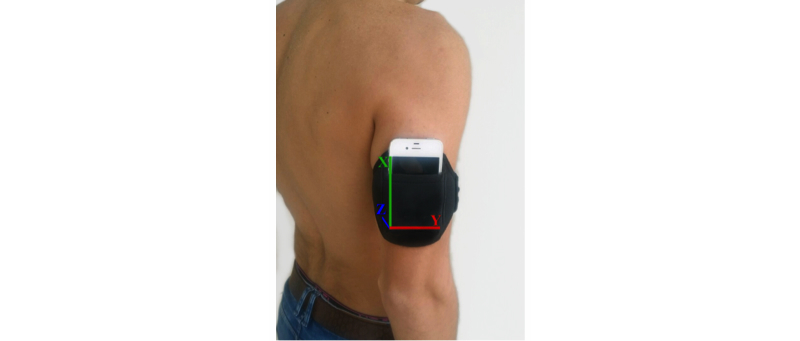
iPhone4 smartphone placed on the right hemi-body of a subject.

Mobility angle (degrees) and acceleration were measured along three orthogonal axes using the iPhone4 (LG Electronics INC, Seoul, South Korea) iOS8.2, which has a storage capacity of 20MB. This phone was placed on the right half of the body of each subject in the triceps skinfold site, located on the posterior part of triceps at mid-acromiale-radiale level (defined by ISAK) [41]. The phone was attached by using a neoprene arm belt (Figure 1) and remained attached throughout. The app used to obtain kinematic data was xSensor Pro (Crossbow Technology, Inc.), available at the Apple AppStore. The data-sampling rate was set to 32 Hz, and the data for each analytical task was transmitted as email for analysis and post-processing. Data from the phone were subsequently sent to a Microsoft Excel 2007 database.

**Figure 2:**
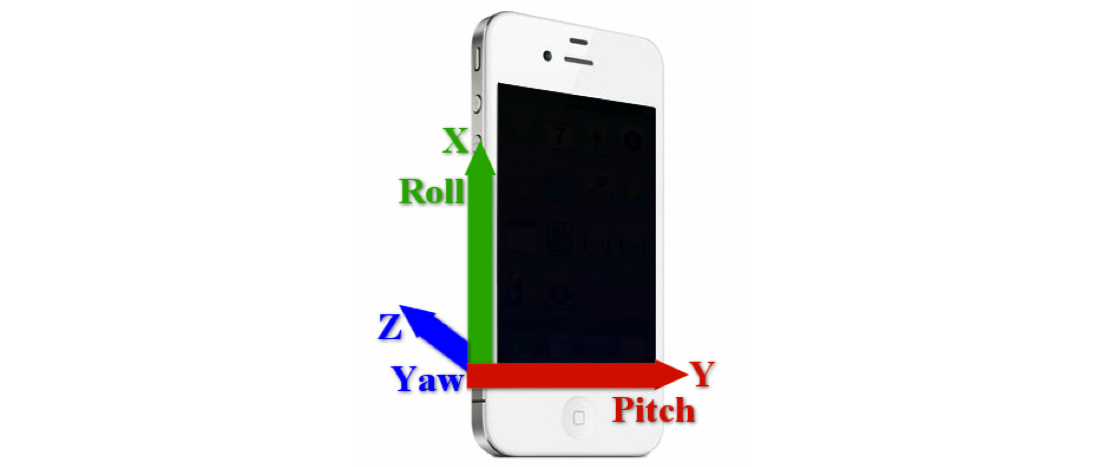
Representation of yaw, pitch, and roll axes in the smartphone placed on humerus.

Because of its positioning, axes and planes in the phone corresponded to different planes of anatomical movement: yaw (z) for shoulder flexor-extension plane, pitch (y) for shoulder abduction plane, and roll (x) for humerus rotation plane (Figure 2).

### Procedure

Subjects were asked to attend the study in the Human Movement Laboratory, Faculty of Health Sciences (University of Málaga). The analytical task to be performed was explained clearly. The beginning and the end were decided by a verbal order by the researcher, which was identical for all participants. They stood, starting from a neutral position, and performed the following analytical tasks: right shoulder abduction for eight repetitions and, after a break of about three minutes, right shoulder flexion for eight repetitions. Participants were told to perform the movements to the highest position they could reach. Both tasks were performed with the elbow extended, the wrist in a neutral position, and the palm area of the hand toward the midline at the beginning and end of the movement.

### Data Analysis

SPSS v15.0 was used for all statistical computations. Descriptive statistics (mean, standard deviation, minimum, and maximum) were calculated for age, height, weight, BMI, angular mobility, and linear acceleration. Standard procedures were used to calculate means and standard deviations. The Kolmogorov-Smirnov test showed a normal distribution of the data (*P*>.05).

Angular mobility and linear acceleration were calculated in two different ways: calculating each space of motion separately and considering the resultant vector of the three axes of movement, which was understood as: Resultant vector = √x^2^+ y^2^+z^2^

## Results

**Figure 3:**
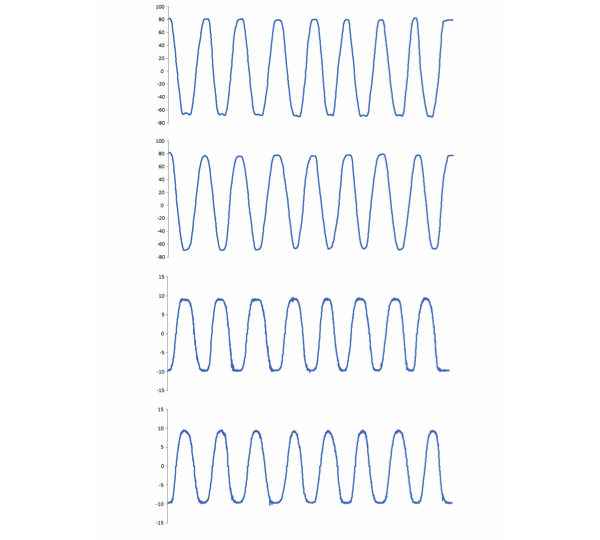
Four examples of kinematic patterns through repetitions shown for angular mobility during ABD (A) and FLEX in pitch axis (B), and the linear acceleration during abduction (C) and flexion (D) in the y-axis.

Analyzing angular mobility allowed us to obtain descriptive graphics of analytical tasks performed by each participant (Figure 3).

Means and standard deviations of angular mobility and acceleration were calculated. For that, data from the second repetition of the second series for both abduction and flexion movements in each of the space axes were analyzed.

In terms of angular mobility, the biggest range was found in pitch axis for flexion movement, followed by the same axis in abduction. However, the smallest range was found in yaw for flexion and in roll for abduction. Considering resultant vector, ROM is bigger for abduction (Table 2).

Regarding acceleration, the largest value was found in the y-axis, followed by the z- and x-axes in both movements. Flexion acceleration was greater in the x-axis when compared to abduction; while, in abduction, acceleration was greater in the y- and z-axes than flex. With regards to resultant vector, acceleration was greater in abduction (Table 3).

Relationship between angular mobility and linear acceleration was calculated for both tasks in each axes of space and resultant vector. Strong correlation was found in y and x as well as in resultant vector, for both tasks. However, that correlation was not significant in yaw axis. More details are shown in Table 4.

**Table 2:** Degrees of angular mobility recorded in abduction and flexion movement.

Angles	Abduction, mean (SD)	Flexion, mean (SD)
Yaw	109.96 (39.44)	79.81 (39.69)
Pitch	151.59 (10.21)	156.15 (12.40)
Roll	87.53 (38.46)	80.09 (47.45)
Resultant vector	209.69 (42.01)	197.89 (42.02)

**Table 3:** Degrees/seconds^2^of acceleration recorded in abduction and flexion movement.

Axes	Abduction, mean (SD)	Flexion, mean (SD)
X	8.48 (1.76)	8.53 (2.8)
Y	19.48 (0.85)	19.43 (0.77)
Z	9.41 (1.5)	7.09 (1.9)
Resultant vector	23.31 (1.58)	22.55 (1.73)

**Table 4:** Pearson correlation between angular mobility and linear acceleration.

Task	Abduction, correlation (*P* value)	Flexion, correlation (*P* value)
Yaw (z)	.462 (.17)	.380 (.2)
Pitch (y)	.914 (<.01)	.915 (<.01)
Roll (x)	.811 (<.01)	.691 (.02)
Resultant vector	.845 (<.01)	.860 (<.01)

## Discussion

### Principal Findings

This study has described and examined upper-limb 3D kinematics using the inertial sensor in the iPhone4 during the performance of shoulder abduction and flexion task in healthy subjects. The biggest range of motion and largest acceleration values varied along each axes. However, taking into account resultant vector from each axes of space, mobility and acceleration were found to be greater for abduction movement. Strong correlation was found between tridimensional mobility and acceleration for both task. The results obtained in this study allow us to obtain descriptive data from upper-limb 3D kinematics, providing an overview of the use of a mobile phone for the study of upper-limb movement.

Previous research has attempted to describe overall upper-limb kinematics through mobile phones. Recently, mobile phone inclinometric measurements of various movements, including abduction and flexion, were performed in 41 affected shoulders. Results showed an acceptable reliability score when compared to conventional goniometers [28]. Very recently, functional assessments of the shoulder through velocity and acceleration inertial variables provided by a mobile phone were studied in five subjects [30]. The use of a clinometer embedded in a mobile phone has been validated in shoulder abduction and flexion movements in healthy and symptomatic shoulders [24]. Recently, mobile phone goniometric measurements have been validated in five healthy subjects, obtaining 95.2° for flexion and 155.4° for abduction [25], which is similar to the abduction degrees obtained in our study. Furthermore, an inclinometery-based and photo-based mobile phone app has been validated for measuring shoulder external rotation [29].

Upper-limb motion has been studied using several devices from decades ago. However, it tends to be deep in kinematic aspects [31] and 3D kinematics [32,42]. For that reason, inertial devices have played an important role when studying shoulder kinematics in several studies [43,44]. Obtaining different results depending on analyzing one plane/axis or its resultant vector intensifies the importance of taking into account the three-dimensional component of anatomical movement, whose analysis is allowed through inertial sensors embedded in mobile phones.

Nowadays, because of new technologies, the concept of telerehabilitation has emerged as an attractive opportunity for provisioning rehabilitation at a distance with the Internet, thus improving the quality of rehabilitation health care [20,21]. Providing comprehensive instructions regarding placement and use of mobile phones would allow patients to measure humerus kinematics, facilitating equitable access to all individuals. Regarding upper-limbs, diagnosis and assessment of musculoskeletal shoulder disorders through the Internet have already been studied [45]. As telerehabilitation is a convenient and easy-to-use system, it would help patients and physicians meet health-related goals. Communication technologies as part of telehealth should also reduce health care costs.

Having reference values of humerus kinematics in the future would be potentially desirable for comparing data from new technologies, such smartphones or smartcameras like Kinect, opening a new world of possibilities in shoulder telehealth assessment.

Tridimensional kinematic tendency, along with the birth of the concept of telerehabilitation, shows the need for mobile phone 3D evaluation of arm movements. The results of this study are in line with other research and show that the use of inertial sensors embedded in mobile phones for upper-limb kinematic analysis appears feasible.

### Limitations

The main weakness of the study is that it is a cross-sectional study, which means cause and effect relationships in kinematic patterns cannot be established. In addition, criterion validity has not been studied because there is no criterion standard.

However, having a sample with a larger number of participants and in which there are also subjects presenting shoulder pathology, we hope to compare our results with those studies reporting on other systems for upper-limb motion analysis. Furthermore, measuring upper-limbs with a gold standard system will allow us to validate mobile phones in upper-limb use as an inertial, easy-to-use measurement. It should be also mentioned that this study estimated only humerus kinematics, while the contribution of other shoulder joints, like sternoclavicular and acromioclavicular ones [46], whose importance has been previously claimed were not included.

### Conclusion

This study discusses humerus kinematics and identifies movement patterns. Therefore, it supports using mobile phones as devices to analyze upper-limb kinematics. Thanks to this study, it is possible to develop a simple and accessible-to-all app that facilitates patient evaluation in this area.

## Abbreviations

BMI: Body Mass Index
ROM: range of motion
3D: three-dimensional
